# Improved cardiorespiratory fitness is associated with functional changes in hippocampus during episodic memory pattern completion

**DOI:** 10.1016/j.nbas.2026.100172

**Published:** 2026-10-14

**Authors:** Emma Simonsson, Micael Andersson, Mattias Hedlund, Erik Rosendahl, Lars Nyberg, Carl-Johan Boraxbekk

**Affiliations:** a Department of Community Medicine and Rehabilitation, Umeå University, Umeå, Sweden; b Umeå center for Functional Brain Imaging (UFBI), Umeå University, Umeå, Sweden; c Department of Medical and Translational Biology, Umeå University, Sweden; d Department of Diagnostics and Intervention, Umeå University, Umeå, Sweden; e Institute for Clinical Medicine, Faculty of Medical and Health Sciences, University of Copenhagen, Copenhagen, Denmark; f Institute of Sports Medicine Copenhagen (ISMC) and Department of Neurology, Copenhagen University Hospital Bispebjerg, Copenhagen, Denmark

**Keywords:** VO_2_max, CA4, DG, Dentate gyrus, Cognition, Older adults

## Abstract

**Background:**

Aerobic exercise that improves cardiorespiratory fitness (CRF) may induce hippocampal changes that help counteract age-related memory decline. The Umeå HIT Study, a randomized controlled trial, examined three months of controlled high-intensity interval training (HIT) and moderate-intensity training (MIT) in older adults. Here, we tested whether increases in CRF (VO₂peak), regardless of training modality, predicted changes in episodic-memory pattern completion and hippocampal structure and function (CA4/DG, CA2/3, CA1).

**Method:**

Forty-two older adults (66–79 years, 45% males) not engaged in regular physical exercise participated in 3 months of twice-weekly supramaximal HIT (20 min including 10 × 6-s intervals) or aerobic MIT (40 min including 3 × 8-m intervals). Before and after the exercise interventions, the participants underwent magnetic resonance imaging (MRI) of the hippocampus, including an episodic memory pattern completion task with concomitant functional MRI.

**Results:**

Exercise-induced change in VO_2_peak (M = 1.5 mL/kg/min, 95% CI[0.8; 2.3]) after three months aerobic exercise of HIT or MIT did not predict change in pattern completion (F_(1,33)_ = 0.02, B = −0.061 *p* = .883, r^2^ = 0.001). However, change in VO_2_peak predicted BOLD signal change in the left hippocampal subfield CA4/DG (F_(1,33)_ = 4.56, B = 0.06 *p* = .04, r^2^ = 0.12), which in turn, predicted change in pattern completion (F_(1,34)_ = 5.19, B = 0.026, *p* = .029, r^2^ = 0.13).

**Conclusion:**

Three months of high- or moderate-intensity training which improves CRF may lead to hippocampal functional changes that, in turn, are linked to enhanced episodic memory pattern completion.

## 1 Introduction

Episodic memory shows a pronounced decline from around the age of 60 [1], is an early predictor of late onset dementia [2], and associated with structural and functional changes of the hippocampus [3], [4], [5], [6], [7], [8]. Older individuals with maintained episodic memory performance show a higher hippocampal activation during task performance compared to average decliners [6]. Thus, understanding the underlying processes essential for episodic memory and potential ways to prevent its decline are important steps for healthy cognitive aging, and ultimately, dementia prevention.

Pattern completion is a hippocampal-dependent process crucial for episodic memory retrieval [9]. It enables stored memories to be recalled from cues containing only partial information of the original memory [10]. This process is more likely to succeed when the cue closely resembles the stored memory, and becomes more challenging and prominent when the resemblance of the incoming information is reduced [10], [11]. It operates in a trade-off with pattern separation, which, in contrast, distinguishes between similar but distinct information. A pattern completion deficit, or a deficit in the trade-off between pattern completion and pattern separation, is proposed to underlie broader cognitive aging and age-related episodic memory decline [12], [13], [14], [15].

The mechanisms underlying a pattern completion deficit in aging remain unclear, but evidence from both animal and human studies supports the idea that different hippocampal subfields play distinct yet complementary roles [16], [17]. Pattern completion has long been associated with the CA3 subfield [9], but it has also been shown that CA1 would be involved in this process [18], [19], [20]. Pattern separation, on the other hand, has been suggested to more rely on the dentate gyrus (DG) in combination with the CA3 [20], [21]. Still, using functional brain imaging, Nyberg et al. [22] observed altered CA4/DG activation among older individuals with impaired pattern completion compared to those with intact pattern completion ability. Further, there is also evidence that most older adults, even those at low risk for neurodegenerative disease, exhibit regional changes in these hippocampal subfields [23].

Notably, higher cardiorespiratory fitness (CRF) has been associated with better episodic memory performance, enhanced pattern separation, and larger hippocampal volumes [24], [25], [26]. Aerobic exercise at moderate intensities that improves CRF has also been linked to better spatial memory, likely mediated by changes in hippocampal blood flow [27] and volume [28], [29]. A recent study in older adults found that higher CRF was positively associated with hippocampal subfield volume in the left CA2/3 and dentate gyrus (DG), and the right CA1 and CA2/3, suggesting both lateralized and subregional specificity in the CRF–hippocampal volume relationship [25]. Similarly, Nauer et al. [30] reported that exercise-induced increases in CRF were associated with increased left DG/CA3 volume in young adults, consistent with rodent and post-mortem human studies on exercise-induced hippocampal neurogenesis [31], [32], [33]. Taken together, aerobic exercise that improves CRF may promote structural and functional brain changes that help mitigate age-related episodic memory decline, potentially through the mechanisms of pattern separation and pattern completion [11], [34], [35].

However, longitudinal, and functional brain imaging data on exercise-induced changes in pattern separation and pattern completion remain sparse. Friedl-Werner et al. [36] examined young adults after 60 days of bed rest combined with plyometric training, compared to bed rest alone, and observed a BOLD signal decrease in the left hippocampus during a pattern separation task but no change in task performance. Among older adults, while higher baseline CRF was associated with better performance in a pattern separation task, exercise-induced improvements in CRF were associated with worsened pattern separation [26]. So, while some research indicates that aerobic exercise benefits episodic memory and pattern separation, findings remain mixed, and our understanding of the underlying pathways of CRF-induced hippocampal changes and their effects on episodic memory remains limited [37], [38]. Further investigation of the CRF-hypothesis at hippocampal subfield level has been requested [25], and to our knowledge, no exercise studies have specifically examined the effects of CRF on pattern completion ability.

In the Umeå HIT Study, a randomized controlled trial, we have previously demonstrated that three months of controlled supramaximal high-intensity interval training (HIT) or aerobic moderate-intensity training (MIT) similarly improved CRF among older adults [39]. In the present study, the aim was to test the hypothesis that the exercise-induced increases in CRF, regardless of training modality, would predict improvements in pattern completion and changes in the underlying structure and function of the hippocampus and its subfields (CA1, CA2/3 and CA4/DG).

## 2 Materials & methods

### 2.1 Study design

The Umeå HIT Study was a randomized controlled trial (RCT) of parallel design with a 1:1 group allocation to controlled supramaximal HIT adapted for older adults [40], or MIT [39]. The study protocol was registered at ClinicalTrials.gov (NCT03765385) and approved by the Regional Ethical Review Board in Umeå (2018–307-31 M, 2018–421-32 M). All participants provided written informed consent and were medically cleared prior to inclusion. In total, 68 non-exercising older adults (i.e., not engaged in any type of regular physical exercise at moderate or high intensity for the past year and with a physical activity score below 11 indicating insufficient physical activity [41], [42]) were recruited. All baseline assessments were completed before randomization to 3-months of exercise with controlled supramaximal HIT or MIT, which was followed directly by 3-month follow-up assessments. The exercise intervention consisted of 25 training sessions performed twice weekly at a local exercise facility in groups of 8 to 10 participants. The supramaximal HIT protocol was 20 min long including ten 6-s intervals, and the MIT protocol was 40 min long including three 8-min intervals. The exercise intensity was watt-controlled and individually progressed throughout the intervention. Detailed descriptions of the main outcomes from the RCT, the training protocols and adherence to the training protocol have previously been published in Simonsson et al. [39] and Frykholm et al. [43].

### 2.2 Participants

Forty-three out of the 68 participants in the Umeå HIT Study underwent structural and functional MRI. Eligible were those without contraindications for MRI scanning (e.g., pacemaker, metal implant, or claustrophobia) and having an eyesight that, if necessary, could be corrected with the scanner compatible glasses. Overall exclusion criteria for the RCT were exercise-induced symptoms of movement-related dysfunction; heart- or lung conditions contraindicative of high-intensity exercise; untreated hypertension (≥ 140/90 mmHg); insulin-treated diabetes; chronic or progressive neurological disease; and a Mini-Mental State Examination score below 27. The MRI was performed by experienced personnel at the University Hospital in Umeå. A radiologist examined all structural images, and no anomalies were detected that required exclusion from the RCT. However, due to a technical error, one participant could not perform the fMRI pattern completion task and was therefore excluded from the present study.

At baseline, the present study included 42 participants with a mean age of 69.7 ± 3 years (45% male), an average of 14.5 ± 3.1 years of education, 76% had some form of medical diagnosis, 60% were diagnosed with high blood pressure and 69% were on some type of medication. Data on CRF, pattern completion, and raw hippocampal volumes at baseline and at 3-month follow-up are presented in Table 1. For the 3-month follow-up, one participant was excluded from MRI as a precaution due to detected superficial metal fragments during the baseline MRI. Further, two participants had voluntarily withdrawn from the overall study, and three participants did not undergo MRI due to illness and subsequent scheduling limitations (separate baseline descriptives in Supplementary Table 1).

**Table 1:** Raw data for CRF, pattern completion and hippocampal volumes at baseline and 3-month follow-up.

	No. ^†^	Baseline	Follow-up
Cardiorespiratory Fitness			
VO_2_peak, mL/kg/min	42/38	23.8 (5.2)	25 (5.4)
VO_2_, L/min	42/38	1.8 (0.4)	1.9 (0.5)
BMI, kg/m^2^	42/39	25.7 (3.2)	25.6 (3.2)

Pattern completion			
Correct recall, range 0–48	42/36	22.7 (5.4)	25.6 (5.9)

Hippocampal volumes (mm^3^)			
Whole Hippocampus	42/36	6392 (617)	6386 (593)
Whole CA1	42/36	1240 (135)	1244 (136)
Whole CA2/3	42/36	417 (46)	412 (48)
Whole CA4/DG	42/36	994 (104)	992 (107)
Left Hippocampus	42/36	3179 (316)	3171 (311)
Left CA1	42/36	607 (69)	608 (69)
Left CA2/3	42/36	199 (25)	198 (26)
Left CA4/DG	42/36	486 (52)	483 (53)
Right Hippocampus	42/36	3213 (314)	3214 (303)
Right CA1	42/36	633 (73)	636 (73)
Right CA2/3	42/36	218 (26)	215 (27)
Right CA4/DG	42/36	508 (56)	509 (59)

*Notes*: Numbers are presented as mean (SD) unless stated otherwise. VO_2_peak = peak oxygen uptake; PC = pattern completion. DG = Dentate Gyrus. ^†^ No. (number) of available participant data at baseline/follow-up.

Twenty-two of the 42 participants were allocated to 3 months of supramaximal HIT and 20 to MIT. Similar to our previous findings [39], [43], the average attendance rate across participants was 89.5% (SD = 8.4) in HIT, and 86% (SD = 22.2) in MIT. Of note, one participant in MIT dropped out from the study without having attended a single exercise session. The two most common reasons for not attending were personal pre-announced (55.9% in HIT, and 53.5% in MIT) or being ill (27.5% in HIT, and 23.8% in MIT). The stipulated median (min; max) interval intensity across all 25 training sessions, as determined from each participant's median, was one minute at 322 W (228; 560 W) in supramaximal HIT and 24 min at 81 W (46; 121 W) in MIT. See Supplementary Table 2 for further numbers on attendance and stipulated training intensity across sessions.

### 2.3 Outcomes and assessments

#### 2.3.1 Pattern completion fMRI task

A detailed description of the fMRI pattern completion task is found in Nyberg et al. [22]. It is a partial cued recall task consisting of 62 items with modified versions of previously presented faces from a face-name task performed outside the scanner (Fig. 1). The face-name task was performed approximately 45 min before the fMRI pattern completion task. There were three different types of items: fragmented images (24 old +4 new), morphed images (24 old +4 new), and 6 old unaltered images. Each item was displayed for 6 s. During the final four seconds the participant was presented with three answer options: the old name of the displayed face, another name previously associated with a different face, and “new face.” The interstimulus interval (ISI) varied randomly between 2.5, 5, and 7.5 s during which a fixation cross (+) was displayed. The participant responded to each item with a button press on a scanner-compatible response pad and was instructed to guess if being unsure. For the pattern completion paradigm, item responses for fragmented and morphed images were defined as correct when the participant responded to an old but altered face with the correct old name (i.e., pattern completion), rather than choosing “new face” (i.e., pattern separation). The outcome measure for pattern completion task performance was the total number of correct responses for old fragmented and morphed images (range 0 to 48).

**Fig. 1:**
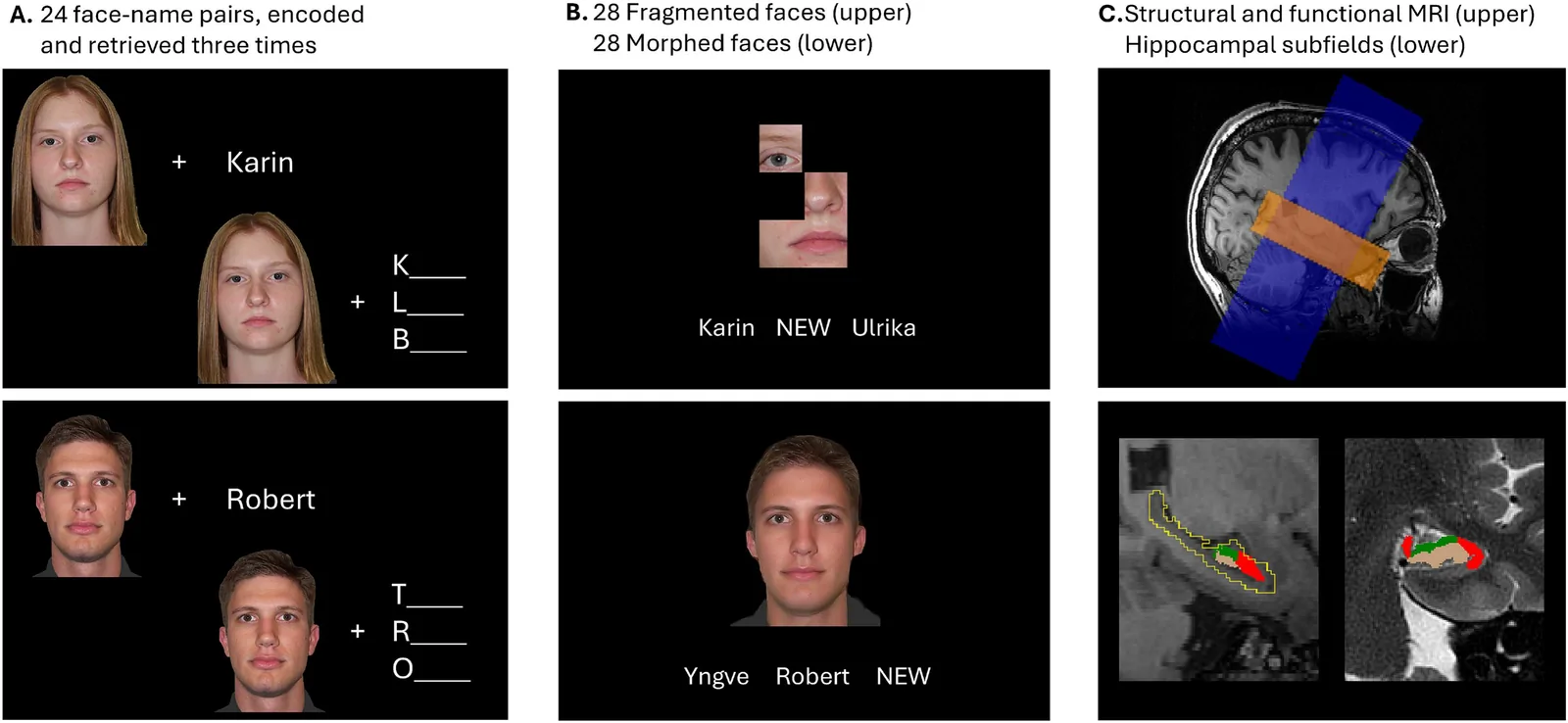
Overview of the episodic memory test of pattern completion and the magnetic resonance imaging (MRI) sequences (Figure modified from Nyberg et al. [22]). Image examples of the (A) face-name task that was performed prior to scanning, and the (B) fragmented and morphed face cues that was used in the in-scanner pattern completion task. (C) The MRI scans included a whole-brain structural T1W-image (grey), a high-resolution structural T2W-image (blue contour), and high-resolution functional volumes (orange) of the hippocampus. Whole-hippocampal subfield segmentation of CA1 (red), CA2/3 (green), and CA4/DG (beige) for the left and right hippocampi was performed with the HippocampalSubfields module in FreeSurfer 7.2. (For interpretation of the references to colour in this figure legend, the reader is referred to the web version of this article.)

#### 2.3.2 MRI acquisition

Structural and functional MRI was obtained with a GE Discovery MR750 3 T equipped with a 32-channel head coil. Structural T1-weighted (T1W) data was acquired in an axial orientation using a 3-dimensional fast spoiled gradient echo sequence (176 slices of 1.0 mm thickness; TR: 8.2 ms; TE: 3.2 ms; TI: 450 ms; flip angle: 12°; field of view: 25 × 25 cm; matrix: 256 × 256 voxels reconstructed to 512 × 512 voxels). High-resolution structural T2-weighted (hr-T2W) data of the hippocampus was acquired in a perpendicular orientation, with respect to the hippocampus long axis (Fig. 1), using a PROPELLER sequence (31 slices of 2.0 mm thickness with no gap; TR: 7312 ms, TE: 11 ms; echo train length: 28; flip angle: 120°; number of excitations: 2.5; field of view: 20.48 cm; matrix: 512 × 512 voxels, resulting in a 0.4 × 0.4 mm in-plane resolution). The B0 field was acquired using a fast gradient echo sequence (B0 map). High-resolution functional MRI (hr-fMRI) data for the hippocampus was acquired in a parallel orientation (Fig. 1), with respect to the hippocampus long axis, using an echoplanar imaging sequence with a reduced field-of-view of the mid part of the brain (20 slices of 1.5 mm thickness with no gap; TR: 1500 ms; TE: 30 ms; flip angle: 75°; field of view: 9.6 × 9.6 cm; matrix: 64 × 64 voxels, resulting in a 1.5 mm isotropic resolution). Ten dummy volumes were acquired (to reach signal equilibrium) and automatically discarded before the acquisition of 470 volumes. Total acquisition time was 12 min.

#### 2.3.3 Structural MRI preprocessing

Hippocampal subfield segmentation was performed with FreeSurfer 7.2 using the HippocampalSubfields module with both the T1W and the hr-T2W images as input, and the CA aggregation combining the CA2 and CA3 (CA2/3) and the CA4 and dentate gyrus (CA4/DG). To create unbiased within-subject template space and extract reliable volume estimates for each time point (baseline and 3-month follow-up), we first performed the longitudinal stream (recon-all -long) with the T1W data as input [44]. Then the T1W-longitudinal segmentation templates were transformed back to native space and used together with the hr-T2W as input for hippocampal subfield segmentation [45]. A quality control of the hippocampal subfield segmentation was based on the ENIGMA Hippocampal Subfields Segmentation Protocols [46], and by comparing the segmentations from combining the longitudinal T1W and hr-T2W images with segmentations using only the hr-T2W images. The most evident issue was that the hr-T2W images did not cover the whole hippocampus tail for all participants, favouring the combined input of T1W and hr-T2W. Also, comparisons of hippocampal volumes (mm^3^) obtained from T1W longitudinal segmentation and hippocampal subfield segmentation (T1W + hr-T2W) showed correlations ranging from 0.81 to 0.87 (Supplementary Fig. 1). T1W longitudinal segmentation tended to produce ~20% larger volumes, possibly due to partial volume effects, further supporting the use of the combined input. In this study, the dependent outcome measures for hippocampal structure were the volumes (mm^3^) of the bilateral, left, and right hippocampus, as well as the hippocampal subfields CA1, CA2/3 and CA4/DG (Fig. 1). All regions were corrected with the intracranial volume estimate from the FreeSurfer segmentation, using the residual method [47], [48].

#### 2.3.4 Functional MRI preprocessing

The hr-fMRI data processing was based on the procedure in Nyberg et al. [22], and the subfield division for structural MRI above. First, because B0-protocol images were offset from other images in native space, an anatomic image from the B0-protocol was co-registered to the T1W image. The resulting transformation matrix was then used to transform the B0 field map to T1W space and generate a voxel displacement map (VDM) used during movement correction to address for B0 field inhomogeneities. Second, the hr-fMRI data was corrected for slice-timing (interleaved order, first image set to reference slice), head movement and B0 field inhomogeneities. Third, the hr-fMRI data was co-registered to T1W space using the transformation matrix from the co-registration during the hippocampus subfield segmentation (T1W + hr-T2W). Fourth, we created single time series (Yb) for each left and right hippocampal subfield (CA1, CA2/3, and DG/CA4) by averaging voxel intensities within each subfield, using masks from the FreeSurfer subfield segmentation (T1W + hr-T2W). The time series were then analysed with a general linear model for each participant, subfield, and time point. A boxcar model with 3 TR for each event, convolved with the standard hemodynamic response function at stimulus onset (when each item-face was displayed). The regressor of interest was correctly recalled old fragmented and morphed faces (CFM), i.e., successful pattern completion. Fragmented and morphed trials were grouped together to achieve a more even distribution of item-responses and increase statistical power for detecting BOLD-signal changes in this event-related design. Five regressors of no interest; incorrectly recalled old fragmented and morphed faces (IFM) (i.e., incorrect old name or “new face”), new fragmented faces (NF), new morphed faces (NM), unaltered faces (U), and fixation cross (FC)). Six realignment parameters (MP) from the motion correction, and a second-order polynomial were also included.

$$\mathrm{Yb}=\beta_{1}\mathrm{CFM}+\beta_{2}\mathrm{IFM}+\beta_{3}\mathrm{NF}+\beta_{4}\mathrm{NM}+\beta_{5}U+\beta_{6}\mathrm{FC}+\beta_{7-12}\mathrm{MP}+\beta_{13}t+\beta_{14}t^{2}+\mu$$

Lastly, for each participant, the obtained beta values for correct answers for the old fragmented and morphed faces during the pattern completion task were normalized as the percent BOLD signal change in relation to the participant's mean of session (MoS). This contrast [correct_answer/MoS * 100] served as the outcome measure for hippocampal pattern completion function and was extracted for the left and right hippocampus, and each hippocampal subfield, respectively.

#### 2.3.5 Cardiorespiratory fitness

Peak oxygen uptake (VO_2_peak) was defined as the highest 30-s mean oxygen uptake (mL/kg/min) measured during a standardized ramp test on a cycle ergometer (Monark 839E Monark Exercise AB, Vansbro, Sweden). The test began at a three-minute constant load of 30 W for females and 40 W for males, followed by a ramp protocol increasing the workload by 10 W/min for females and 15 W/min for males. Expired air was continuously analysed during the test using a mixing chamber-based ergo-spirometry system (AMIS 2001 Innovision A/S, Odense, Denmark). The test was terminated at volitional exhaustion, most commonly corresponding to a perceived exertion of 18 or higher on the Borg Rating of Perceived Exertion scale [49], or according to predefined safety criteria. See Supplementary Methods for further details on test procedures and test termination criteria.

### 2.4 Blinding

The participants were informed about the general study purpose but blind to study hypotheses and only received detailed information about their allocated exercise group. The participants were repeatedly instructed to withhold any details of their group allocation during the follow-up assessments. All outcome assessors were blinded to group allocation, except E.S. at the 3-month follow-up MRI assessments. E.S. administered the cognitive tests included in the MRI session. In cases of overlapping schedules, E.S administered the outside-scanner introduction and practice rounds, while a cognitive neuroscience student administered the in-scanner task.

### 2.5 Statistical methods

The primary analyses of this paper were conducted in R [50] and RStudio [51], using the tidyverse [52], lme4 [53], emmeans [54], and effectsize [55] packages. Linear mixed-effects models (LMMs) were used to examine overall changes over time (baseline and follow-up values) for CRF, pattern completion, hippocampal structure (volume) and function (BOLD). For each outcome, the model included group (MIT as reference), age (years), sex (male/female), and education (years) as fixed effects covariates of no interest, and individual as a random effect (intercept only). LMM-results are reported as the model-estimated mean change with a 95% confidence interval (95% CI) (Supplementary Table 3). Given the focus on individual differences in intervention-related change, results are reported only for the primary predictors of interest. For the primary cognitive outcome (pattern completion performance), the full model output including covariate estimates is provided (Supplementary Table 4). To examine individual differences in intervention responsiveness, linear regressions using change scores (Δ baseline-to-follow-up values) were conducted to assess whether changes in CRF were associated with changes in pattern completion and structure/function of the hippocampus and its subfields (CA1, CA2/3, and CA4/DG). For any significant associations between CRF and hippocampal volume or function, additional analyses were conducted using the same linear regression approach to examine whether changes in specific hippocampal regions predicted changes in pattern completion performance. The results are reported with F-statistics (F), *p*-values (p), and coefficients of determination (R^2^). An α < 0.05 was chosen as indication of statistical significance.

## 3 Results

After three months of aerobic exercise of controlled supramaximal HIT or MIT, the participants in the present study had significantly improved their CRF measure as VO_2_peak (M = 1.5 mL/kg/min, 95% CI [0.8; 2.3]), and their episodic memory pattern completion measured as the total number of correctly recalled fragmented and morphed faces (M = 2.9, 95% CI [1.2; 4.7]). Consistent with our previous findings [39], the present study sample showed no significant between-group differences in VO₂peak changes when comparing supramaximal HIT with MIT (MD = 0.7 mL/kg/min, 95% CI [−0.8; 2.2]). Likewise, we observed no significant between-group differences in changes in pattern completion scores (MD = 1.4, 95% CI [−2.0; 4.8]). Based on these results, we combined the two exercise groups for all subsequent analyses of change–change associations, focusing on individual CRF responsiveness rather than training modality.

The improved CRF, i.e., change in VO_2_peak (mL/kg/min) did not predict change in episodic memory patter completion (F_(1,33)_ = 0.02, B = −0.061 *p* = .883, r^2^ = 0.001) (Fig. 2A), nor did it predict change in any of the hippocampal volumes which included the bilateral, left and right total hippocampus and the CA1, CA2/3 and CA4/DG subfields (Supplementary Fig. 2). For the regression models assessing whether changes in VO_2_peak predicted changes in hippocampal function (Supplementary Fig. 3), measured as BOLD signal change during correctly recalled old fragmented and morphed faces, the only significant association was observed for the left CA4/DG subfield (F_(1,33)_ = 4.56, B = 0.06 *p* = .04, r^2^ = 0.12), with change in VO_2_peak explaining approximately 12% of the variance in activation change (Fig. 2B). In turn, the BOLD signal change in left CA4/DG region was positively associated with change in correctly recalled old fragmented and morphed faces for the pattern completion task (F_(1,34)_ = 5.19, B = 0.5.09, *p* = .029, r^2^ = 0.13), with the left CA4/DG BOLD signal change explaining approximately 13% of the variance in performance change (Fig. 2C). Overall, on group level, there were no significant changes in left CA4/DG BOLD signal (M = −0.01, 95% CI [−0.13; 0.11]) or volume (M = −2.1 mm^3^, 95% CI [−8.75; 4.58]) after the three months of exercise, or any other subfields (Supplementary Table 3).

**Fig. 2:**
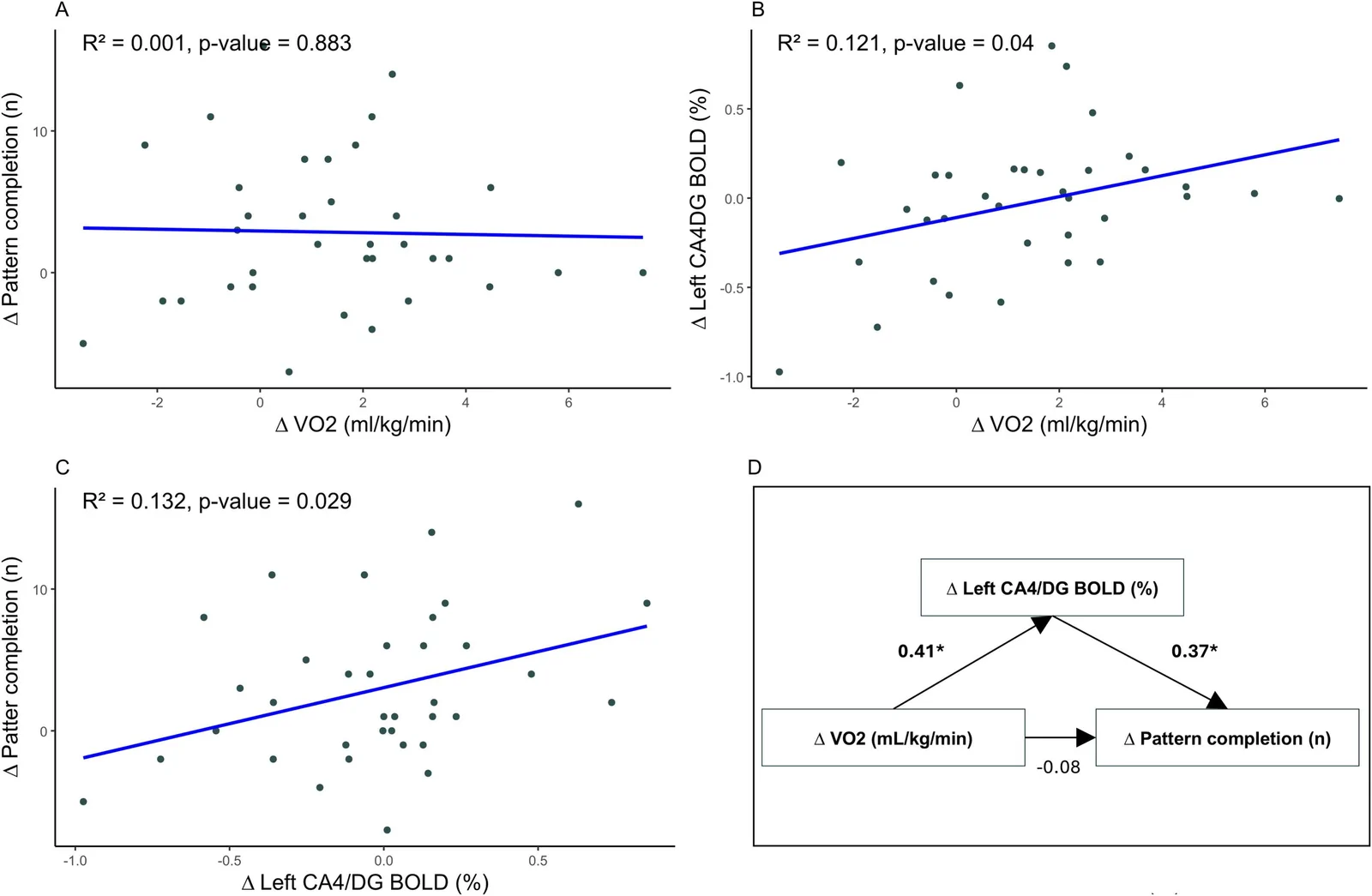
Scatterplots with change–change linear regressions between A) change in cardiorespiratory fitness (∆VO_2_ in mL/kg/min) and change in number of correct pattern completion responses ((∆Pattern completion); B) change in cardiorespiratory fitness and change in hippocampal subfield function (∆ BOLD in %) during the in-scanner pattern completion task for the left CA4/DG; and C) change in left CA4/DG BOLD and change in pattern completion. D) Post hoc structural equation model examining left CA4/DG BOLD signal change as a mediator of the relationship between changes in VO₂ and pattern completion, controlling for age, sex, and education. Standardized coefficients shown and * = *p* < .05.

An exploratory post hoc path analysis was conducted to examine whether BOLD activation mediated the relationship between VO₂ and pattern completion, while controlling for age, sex, and education (Fig. 2D). Change in VO₂peak was still positively associated with change in left CA4/DG BOLD activation (std. β = 0.41, *p* = .017), which in turn was associated with pattern completion performance (std. β = 0.37, *p* = .046). The direct effect of VO₂ on pattern completion was not significant (β = −0.085, *p* = .593), and neither the indirect effect (std. β = 0.15, *p* = .131) nor the total effect (std. β = 0.065, *p* = .680) reached statistical significance. Results remained essentially the same when accounting for baseline fitness differences using revisualized change in VO₂peak. In this model the association between VO₂peak and left CA4/DG BOLD activation remained significant (std. β = 0.43, *p* = .012), while the association between CA4/DG BOLD and pattern completion was attenuated (std. β = 0.36, *p* = .06). The direct (std. β = −0.06, *p* = .738), indirect (std. β = 0.15, *p* = .136), and total effect (std. β = 0.10, *p* = .541) remained non-significant. However, both models were just-identified, precluding assessment of global fit (χ^2^(0) = 0.00, CFI = 1.00, RMSEA = 0.00).

## 4 Discussion

In this study, we examined whether exercise-induced changes in CRF would predict changes in episodic memory pattern completion via underlying structural and functional changes in the hippocampus. After three months of controlled supramaximal HIT or MIT we observed small improvements in CRF and episodic memory pattern completion, with no significant between-group differences. While there was no direct association between change in CRF and change in pattern completion, there was a positive association between change in CRF and left CA4/DG BOLD signal change during successful pattern completion. In turn, the left CA4/DG BOLD signal change was positively associated with the change in correct responses during the pattern completion task, providing partial support for our hypotheses. However, although the path analysis indirect estimate was in the hypothesized direction, it was imprecise and therefore remains inconclusive. Future studies with larger samples are needed to conclude whether a mediation effect is present.

### 4.1 Pattern completion, fMRI activation and cardiorespiratory fitness

Pattern completion, a hippocampal-dependent process crucial for episodic memory retrieval [9], involves distinct yet complementary roles of different hippocampal subfields [16], [17]. Although traditionally linked to the CA3 and CA1 subfields [9], [18], [19], [20], we found that exercise-induced change in CRF was associated with pattern completion BOLD signal change in the left CA4/DG. In turn, increased activity was associated with increased pattern completion performance. Although the DG has previously been associated with pattern separation, its computations are tightly coupled to those of CA3 and enhanced DG integrity has been proposed to help restore the circuit balance required for effective pattern completion [15], [56], [57]. Accordingly, the present results linking CA4/DG activity to pattern completion performance may reflect improved neural efficiency or healthier input sparsity that ultimately facilitates CA3's auto-associative retrieval.

However, in the absence of a non-exercising control-group, we cannot fully rule out that the observed improvement in pattern completion is due to test-enhanced learning effects unrelated to the exercise interventions. That said, a learning effect alone would not explain the observed positive association between changes in CRF and the left CA4/DG BOLD signal, suggesting that our result is at least in part physiologically driven. A previous study in young adults observed a BOLD signal decrease in the left hippocampus, but no change in pattern separation task performance, after 60 days of bed rest combined with plyometric training, compared to controls [36]. That study used different sets of images during the pre- and post-assessments of pattern separation to control for test-retest learning effects. While their observed decrease in left hippocampal BOLD signal may reflect increased neural efficiency for the specific task demand [36], the association between left CA4/DG BOLD signal change and change in pattern completion in our study may reflect increased neural activity due to enhanced memory representations.

This is in line with previous studies comparing hippocampal activation between individuals with high and low episodic memory performance, where high-performers exhibited a stronger functional activation [6], [22]. Notably, in Nyberg et al. [22], older individuals with impaired pattern completion performance showed altered CA4/DG activation for noisier cues (i.e., morphed images) compared to those with intact pattern completion ability. Specifically, their cross-sectional data showed that participants categorized as intact displayed a stronger CA4/DG signal for correctly identified old images, while the impaired group showed a reversed relationship [22]. In the present study, the observed associations between change in CRF, left CA4/DG BOLD signal, and pattern completion performance, may thus represent one underlying pathway of CRF-induced hippocampal changes and subsequent effects on episodic memory in older individuals. Higher CRF has been associated with better pattern separation accuracy at baseline in both young and older adults, however, the results differ regarding exercise-induced changes in CRF and its association with changes in pattern separation [26], [30]. Among lower-fit young adults (< 36 mL/kg/min at baseline), Nauer et al. [30] observed a positive relationship between changes in CRF and pattern separation accuracy during the noisiest cues. Sokołowski et al. [26] on the other hand suggested a potentially inverse relationship among older adults. Yet, without accompanying functional brain imaging data, it is difficult to draw conclusions about the underlying mechanisms behind these diverging findings [58].

Our observed positive associations between improved CRF and BOLD signal change in the left CA4/DG, and between the left CA4/DG BOLD signal change and pattern completion are in line with the CRF-hypothesis, i.e., that enhanced CRF should drive improved cognitive performance through exercise-induced changes in hippocampal function [11], [59]. It further adds a functional component (BOLD) to the causal models proposed by Maass et al. [27] suggesting that CRF-induced changes in episodic memory are directly linked to changes in perfusion. Notably, in the Maass et al. [27] perfusion model, a volumetric change was not necessarily required to achieve improved memory, which our BOLD-data also supports. Thus, while we did not observe any volumetric associations with CRF, we suggest that improved behavioural performance is associated with increased functional activity.

It should be noted that while we did not observe a direct association between changes in CRF and episodic memory pattern completion after 12 weeks of exercise, our participants only increased about 5% in VO_2_peak. Within the same time span, the study by Maass et al. [27], which observed a positive association between changes in CRF and episodic memory object recognition, reported a 10% improvement in ventilatory threshold VO_2_. Similar to other studies (e.g., [27], [29]) we also observed large individual differences in response [39]. Potential reasons for individual differences in response have recently been highlighted. For example, baseline white matter hyperintensities was proposed as one limiting factor for exercise-induced brain plasticity [60]. Additionally, baseline differences in physical function, fitness, as well psychological factors have all been linked with the long-term relationship of physical function and brain health in aging [61]. Thus, identifying these factors will be key to developing more effective and personalized interventions for healthy aging.

### 4.2 Cardiorespiratory fitness and hippocampal volumes

While not evident in our study, others have reported positive effects of exercise on hippocampal volume [28], [30], [62]. Following a one-year aerobic exercise intervention among older adults, Erickson et al. [28] reported a 2% increase in anterior hippocampal volume, alongside a positive association between exercise-induced changes in CRF (+7.8% in VO_2_max) and hippocampal volume. Additionally, they observed a positive relationship between changes in hippocampal volume and improvements in episodic memory (spatial recognition), although no direct association between changes in CRF and spatial recognition was found. In comparison, Maass et al. [27] found a positive association between improved CRF and hippocampal head volume after 12 weeks of aerobic exercise, and Jonasson et al. [29] observed a positive association between improved CRF and total hippocampal volume after 6 months of aerobic exercise. However, neither study found a clear difference in volume changes between groups over time. At the subfield level, Nauer et al. [30], reported that change in CRF was positively associated with change in the left CA3/DG volume after 12 weeks of aerobic exercise in young adults. Notably, while they also observed a baseline association between CRF and the left CA3/DG head in young adults, and therefore suggested that exercise-induced volumetric changes in the hippocampus may be specific to the anterior hippocampus and particularly the CA3/DG subfields [30], this was not replicated in a sample of older adults, where instead, an association was found between baseline CRF and the left CA3/DG body [63]. Together, these findings suggest that there may not only be subfield-specific but also age-specific differences in the relationship between CRF and hippocampal structure, both at the level of subfield volumes and along the hippocampal longitudinal axis (i.e., head, body, and tail). These possibilities warrant further investigation.

### 4.3 Strengths and limitations

Strengths of this study include the multimodal assessments of the effects of aerobic exercise on episodic memory, combining peak oxygen uptake (VO_2_peak) measured with breath-by-breath analysis of inspired and expired gases during incremental exercise, high-resolution structural MRI of hippocampal subfields, and high-resolution task-based functional MRI during a pattern completion paradigm. Furthermore, while relatively few intervention studies have simultaneously examined exercise-related changes in episodic memory together with both structural and functional hippocampal imaging, the present study design allowed us to investigate potential underlying neural mechanisms associated with changes in CRF after three months of individualized supramaximal HIT and MIT on pattern completion performance [38].

We note that the observed effects were generally small, both in terms of CRF improvements and the relationship with functional imaging data. Importantly, these modest effects were observed despite the use of gold-standard physiological assessments and high-resolution neuroimaging methods, suggesting that any exercise-related influences on hippocampal function and pattern completion may be subtle in magnitude within the present sample and intervention period.

Furthermore, the results did not survive correction for multiple comparisons and should therefore be interpreted cautiously until replicated in larger studies. Although correction for multiple comparison correction is important for controlling false-positive findings [64], such procedures may also be overly conservative, potentially masking meaningful and true effects in studies with relatively small sample sizes [65]. Importantly, all hippocampal subfields included in the analyses were specified a priori based on previous work by Nyberg et al. [22], which strengthens the interpretability of the observed patterns despite the lack of significance after multiple-comparison correction.

While even just one training session have shown to induce small but significant acute changes in hippocampal microstructure and memory performance ([66], [67]; [68]), a longer time span and use of more than two repeated measures would be needed to determine the timing of different changes in relation to each other and whether exercise-induced structural effects on the hippocampus leads to memory improvements [62], [69]. Given that exercise-induced physiological changes are proposed to precede changes in cognitive function [11], [59], future studies investigating the neural mechanisms underlying exercise-related cognitive changes should ideally ensure greater physiological effects. In this context, longer intervention durations may be particularly worth considering. Training session intensity and/or weekly session frequency may also be adjusted to enhance the physiological effects of the intervention. However, among older adults undertaking very high-intensity training, interventions should ensure adequate recovery between sessions [70], [71], as well as account for individual readiness by allowing flexibility in the time required to progressively increase training intensity across sessions [43].

In addition, while it is possible that longer intervention durations and larger improvements in CRF are required to detect significant volumetric changes [62], direct comparisons across present studies are complicated not only because of differences in duration and effects on CRF, but also due to differences in assessment methods. For example, we observed a 5% increase in VO₂peak after 12 weeks of exercise, whereas others have reported a 7.4% increase in submaximal estimation of VO_2_max after 12 weeks in young adults [30], a 10% increase in VO₂ at the ventilatory threshold after 12 weeks in older adults [27], and a 7.8% increase in VO₂max after one year in older adults [28].

Another important note of caution is that the majority of the studies mentioned above, including ours, have relied on 3 T MRI scanners for hippocampal subfield segmentation. While this is a common approach and we applied high-resolution imaging sequences, 7 T MRI may be preferable due to its superior spatial resolution and more precise delineation of subfield boundaries [72], [73]. Additionally, substantial variability in the resolution of both structural and functional MRI acquisition protocols, the use of cross-sectional versus longitudinal processing pipelines, as well as hippocampal subfield segmentation and aggregation methods further complicates direct comparisons across studies [11], [46], [74].

The small effects observed in the present study could further be put in relation to the overall small changes in CRF observed in the main trial [39], the generally small effects that have been presented for the link between CRF, hippocampus and episodic memory [25], [75], as well as for exercise-induced changes in the hippocampus and episodic memory [29], [37], [62]. Nevertheless, as we age, small changes may have a significant impact and to our knowledge this is one of the few studies that have incorporated repeated measures of both CRF, structural and functional MRI with a concomitant episodic memory test to assess the effects of aerobic exercise on the hippocampus and episodic memory pattern completion.

## 5 Conclusion

Increased cardiorespiratory fitness following three months of either supramaximal high-intensity interval training (HIT) or moderate-intensity training (MIT) was associated with hippocampal functional activity changes in the left CA4/DG during successful pattern completion, which, was further associated with improved performance in pattern completion. Future studies should further investigate the mechanisms driving exercise-related changes in cognition and the factors that contribute to substantial individual differences in responsiveness to exercise interventions, in larger and adequately powered samples, to better inform the development of personalized and effective strategies for brain health in aging populations.

## CRediT authorship contribution statement

**Emma Simonsson:** Writing – review & editing, Writing – original draft, Visualization, Project administration, Methodology, Investigation, Funding acquisition, Formal analysis, Data curation. **Micael Andersson:** Writing – review & editing, Formal analysis, Data curation. **Mattias Hedlund:** Writing – review & editing, Project administration, Investigation, Conceptualization. **Erik Rosendahl:** Writing – review & editing, Supervision, Funding acquisition, Conceptualization. **Lars Nyberg:** Writing – review & editing, Resources, Methodology. **Carl-Johan Boraxbekk:** Writing – review & editing, Supervision, Resources, Methodology, Funding acquisition, Conceptualization.

## Declaration of competing interest

The authors declare no known competing financial interests or personal relationships that could have appeared to influence the work reported in this paper.
