# Supplementary material for: Improved cardiorespiratory fitness is associated with functional changes in hippocampus during episodic memory pattern completion
Source: Aging Brain. 2026 Oct 14;10:100172. doi: 10.1016/j.nbas.2026.100172 (PMC13694721; doi:10.1016/j.nbas.2026.100172)
Supplement: Supplementary file 1 [file mmc1.docx]

# Supplementary Files

## Supplementary Methods

**2.3.5 Cardiorespiratory Fitness**

Test termination criteria:

1) volitional exhaustion
2) inability to maintain a cadence of 60 rpm despite encouragement
3) heart rate and/or V̇O2 plateau despite increased intensity
4) RPE exceeding 17 (Borg, 1998)
5) test leader judging the participant as too exhausted
6) any perceived chest pain (CR10-scale exceeding 0, (Borg, 1998))
7) drop of systolic blood pressure of 15 mmHg or more, or systolic blood pressure above 250 mmHg.

A Polar H10 chest belt (Polar Electro Oy, Kempele, Finland) continuously measured the participant’s heart rate. Blood pressure was measured at rest, every second minute during the test, immediately after the test, as well as 2 and 4 minutes after termination of the test. Self-perceived exertion was assessed with the 15-point (6-20) Borg Rating of Perceived Exertion scale (RPE) at the end of the first three minutes and then by every other following minute. When the test leader judged the participant to be close to end/termination, RPE was assessed at a closer interval. The Borg Category Ratio (CR-10) scale was assessed using the same procedure as the Borg Scale above, immediately after the participant was asked about RPE. All participants were instructed to rate the strength of any unpleasant sensation from the chest i.e., chest pain, according to the scale. Lactate was assessed at rest prior to start and immediately after test termination.

References:

Borg, G. (1998). *Borg’s perceived exertion and pain scales* (pp. viii, 104). Human Kinetics.

## Supplementary Table 1

| **Supplementary Table 1.** Baseline descriptives and raw data for CRF, pattern completion and hippocampal volumes at baseline for all participants, complete cases (baseline and follow-up), and cases only providing data at baseline. | | | |
| --- | --- | --- | --- |
|  | All  (n = 42) | Complete cases  (n = 36) | Cases with baseline data only (n = 6) |
| Baseline characteristics |  |  |  |
| No. males (%) | 19 (45) | 15 (42) | 4 (67) |
| Age, years | 69.7 (3) | 69.3 (2.7) | 72.2 (3.7) |
| Education, years | 14.5 (3.1) | 14.5 (3) | 14.7 (3.6) |
| Cardiorespiratory Fitness |  |  |  |
| VO_2_peak, mL/kg/min | 23.8 (5.2) | 23.9 (5.5) | 23.4 (4.1) |
| VO_2_, L/min | 1.8 (0.4) | 1.8 (0.4) | 1.9 (0.5) |
| BMI, kg/m^2^ | 25.7 (3.2) | 25.6 (3.3) | 26.3 (3.1) |
| Pattern completion |  |  |  |
| Correct recall, range 0-48 | 22.7 (5.4) | 22.6 (5.5) | 23.5 (4.6) |
| Hippocampal volumes (mm^3^) |  |  |  |
| Whole Hippocampus | 6392 (617) | 6413 (623) | 6264 (615) |
| Whole CA1 | 1240 (135) | 1243 (137) | 1225 (132) |
| Whole CA2/3 | 417 (46) | 416 (47) | 421 (48) |
| Whole CA4/DG | 994 (104) | 990 (105) | 1016 (106) |
| Left Hippocampus | 3179 (316) | 3188 (324) | 3121 (281) |
| Left CA1 | 607 (69) | 606 (72) | 613 (51) |
| Left CA2/3 | 199 (25) | 200 (25) | 196 (22) |
| Left CA4/DG | 486 (52) | 485 (53) | 490 (50) |
| Right Hippocampus | 3213 (314) | 3225 (314) | 3142 (336) |
| Right CA1 | 633 (73) | 637 (71) | 612 (83) |
| Right CA2/3 | 218 (26) | 217 (26) | 225 (27) |
| Right CA4/DG | 508 (56) | 505 (56) | 526 (57) |
|  | | | |
| *Notes*: Numbers are presented as mean (SD) unless stated otherwise. VO_2_peak = peak oxygen uptake; PC = pattern completion. DG = Dentate Gyrus. | | | |

## Supplementary Table 2

| Supplementary Table 2. Overview of participant exercise-session attendance and stipulated training intensity for the first, last and median session for supramaximal HIT and MIT. | | |
| --- | --- | --- |
|  | Supramaximal HIT (n = 22) | MIT  (n = 20) |
| **Session attendance** |  |  |
| Overall, n (%) | 492 (89.5) | 430 (86) |
| Participant, median (min; max) | 22 (18; 25) | 23 (0; 25) |
| **Stipulated interval intensity** |  |  |
| First session, median (min;max) |  |  |
| - in watts (W) | 194 (155; 336) | 60 (37; 87) |
| - in % of MPO6 | 60 | - |
| - in % of MAP | 171 (131; 241) | 40 |
| Last session, median (min;max) |  |  |
| - in watts (W) | 392 (321; 605) | 80 (44; 130) |
| - in % of MPO6 | 110 (96; 136) | - |
| - in % of MAP | 327 (264; 442) | 66 (40; 90) |
| - total work (kJ) | 64 (46; 107) | 160 (99; 250) |
| Median session, median (min;max) |  |  |
| - in watts (W) | 322 (228; 560) | 81 (46; 121) |
| - in % of MPO6 | 92 (72; 100) | - |
| - in% of MAP | 251 (188; 374) | 63 (45; 85) |
| - total work (kJ) | 61 (45; 102) | 161 (94: 237) |
|  | | |
| *Notes*. The supramaximal HIT protocol was 20 minutes long including ten 6-second intervals, and the MIT protocol was 40 minutes long including three 8-minute intervals. The training intensity was watt-controlled and individually progressed throughout the intervention. Abbreviations: HIT = high-intensity interval training; MIT = moderate-intensity training; MPO6 = maximum mean power output for 6 seconds estimated at baseline testing; MAP = maximum aerobic power at baseline testing of peak oxygen uptake. | | |

## Supplementary Table 3

Change from Baseline to Follow-Up for Hippocampus Structure and Function, for bilateral, left, and right hippocampus and the hippocampal subfields CA1, CA2/3, and CA4/DG.

| **Supplementary Table 3. Change from Baseline to Follow-Up for Hippocampus Structure and Function** | | | | | |
| --- | --- | --- | --- | --- | --- |
|  | | | | | |
| **Outcomes** | **No.** ^†^ | **Mean** | **[95% CI]** | **p** ^‡^ | **ES** |
| **Cardiorespiratory fitness** |  |  |  |  |  |
| VO_2_peak (mL/kg/min) | 42 / 38 | 1.52 | [0.77; 2.25] | .0002 | 0.28 |
| VO_2_, L/min | 42 / 38 | 0.1 | [0.05; 0.16] | .001 | 0.23 |
| BMI, kg/m^2^ | 42 / 39 | -0.17 | [-0.29; -0.05] | .009 | -0.05 |
| **Pattern completion** |  |  |  |  |  |
| Correct recall (n) | 42 / 36 | 2.94 | [1.22; 4.65] | .002 | 0.52 |
| **HC structure (mm^3^)** |  |  |  |  |  |
| Whole HC | 42 / 36 | -26.47 | [-67.31; 14.88] | .419 | -0.06 |
| CA1 | 42 / 36 | 0.78 | [-8.86; 10.5] | .874 | 0.01 |
| CA2/3 | 42 / 36 | -3.86 | [-8.16; 0.46] | .337 | -0.09 |
| CA4/DG | 42 / 36 | 1.81 | [-7.35; 10.96] | .874 | 0.02 |
| Left HC | 42 / 36 | -15.67 | [-43.62; 12.71] | .581 | -0.06 |
| CA1 | 42 / 36 | 1.72 | [-5.29; 8.74] | .72 | 0.03 |
| CA2/3 | 42 / 36 | -1.75 | [-5.33; 1.91] | .581 | -0.07 |
| CA4/DG | 42 / 36 | -2.1 | [-8.75; 4.58] | .714 | -0.05 |
| Right HC | 42 / 36 | -10.33 | [-32.45; 12.06] | .581 | -0.04 |
| CA1 | 42 / 36 | -0.9 | [-6.59; 4.85] | .755 | -0.02 |
| CA2/3 | 42 / 36 | -2.01 | [-4.89; 0.85] | .581 | -0.08 |
| CA4/DG | 42 / 36 | 3.96 | [-1.12; 9.01] | .581 | 0.08 |
| **HC function (BOLD)** |  |  |  |  |  |
| Whole HC | 42 / 36 | -0.05 | [-0.1; 0] | .135 | -0.31 |
| CA1 | 42 / 36 | -0.04 | [-0.12; 0.05] | .543 | -0.18 |
| CA2/3 | 42 / 36 | -0.1 | [-0.18; -0.02] | .08 | -0.44 |
| CA4/DG | 42 / 36 | 0.002 | [-0.08; 0.09] | .955 | 0.01 |
| Left HC | 42 / 36 | -0.05 | [-0.12; 0.02] | .33 | -0.24 |
| CA1 | 42 / 36 | 0.01 | [-0.1; 0.12] | .858 | 0.04 |
| CA2/3 | 42 / 36 | -0.11 | [-0.25; 0.04] | .33 | -0.32 |
| CA4/DG | 42 / 36 | -0.01 | [-0.13; 0.11] | .858 | -0.04 |
| Right HC | 42 / 36 | -0.06 | [-0.11; 0] | .33 | -0.29 |
| CA1 | 42 / 36 | -0.08 | [-0.17; 0.03] | .33 | -0.32 |
| CA2/3 | 42 / 36 | -0.09 | [-0.21; 0.04] | .33 | -0.29 |
| CA4/DG | 42 / 36 | 0.01 | [-0.09; 0.12] | .858 | 0.05 |
|  | | | | | |
| *Notes.* Estimates of mean change from linear mixed-effects models (LMM) adjusted for fixed (exercise group (moderate-intensity training group as reference), age, sex (female as reference), and education) and random intercept (individual) effects, together with 95% CI. Effect Size (ES) was calculated from the model estimate of mean change and the unadjusted pooled standard deviation. Abbreviations: HC = Hippocampus, BOLD = Blood-Oxygen-Level-Dependent.  ^†^ No. (number) of available participant data at baseline / follow-up.  ^‡^ Analyses of Hippocampal structure and function are corrected for multiple comparisons and presented with false discovery rate (FDR) corrected p-values (Benjamini & Hochberg, 1995). | | | | | |

## Supplementary Table 4

| **Supplementary Table 4. LMM Fixed Effects Model Output for the Primary Cognitive Outcome Pattern Completion (correct recall, n)** | | | | | | |
| --- | --- | --- | --- | --- | --- | --- |
|  | | | | | | |
| **Term** | **Estimate (β)** | **[95% CI]** | **SE** | **df** | **t** | **p** |
| **Fixed effects** |  |  |  |  |  |  |
| **(Intercept)** | 18.4 | [10.5; 26.31] | 4.20 | 38.0 | 4.387 | >.001 |
| **Primary longitudinal effect** |  |  |  |  |  |  |
| Visit (follow-up) | 2.94 | [1.22; 4.65] | 0.87 | 37.9 | 3.389 | .002 |
| **Covariates** |  |  |  |  |  |  |
| Age (years from 65) | -0.0005 | [-0.49; 0.49] | 0.26 | 40.2 | -0.002 | .999 |
| Sex (Male) | -1.46 | [-4.36; 1.45] | 1.54 | 37.8 | -0.945 | .351 |
| Education (years) | 0.23 | [-0.25; 0.71] | 0.26 | 37.9 | 0.900 | .374 |
| Exercise group (HIT) | 3.16 | [0.31; 6.01] | 1.51 | 37.7 | 2.089 | .043 |
|  |  |  |  |  |  |  |
| *Notes.* Estimated marginal means of linear mixed-effects models of the study’s primary cognitive outcome adjusted for fixed (exercise group (moderate-intensity training group as reference), age, sex (female as reference), and education) and including a random intercept for individuals, together with 95% CIs, standard errors (SE), degrees of freedom (df), t-values, and p-values. | | | | | | |

## Supplementary Figure 1

Linear regressions for baseline whole (A), left (B) and right (C) hippocampus volumes (mm^3^) from FreeSurfer 7.2 longitudinal whole-brain segmentation (x-axis) using T1-weighted (T1W) images and hippocampal subfield segmentation (y-axis) using T1-weighted + high-resolution T2-weighted images. T1W longitudinal segmentation tended to produce ~20% larger volumes, possibly due to partial volume effects.

## Supplementary Figure 2

Scatterplots with linear regressions of change–change associations between change in cardiorespiratory fitness (∆VO_2_ in mL/kg/min) and changes in hippocampal volume (mm^3^) for bilateral, left, and right hippocampus and the hippocampal subfields CA1, CA2/3, and CA4/DG.

## Supplementary Figure 3

Scatterplots with linear regressions of change–change associations between change in cardiorespiratory fitness (∆VO_2_ in mL/kg/min) and changes in hippocampal function (BOLD %) for bilateral, left, and right hippocampus and the hippocampal subfields CA1, CA2/3, and CA4/DG
